# Development of 3D Force Sensors for Nanopositioning and Nanomeasuring Machine

**DOI:** 10.3390/s90503228

**Published:** 2009-04-28

**Authors:** Arti Tibrewala, Norbert Hofmann, Anurak Phataralaoha, Gerd Jäger, Stephanus Büttgenbach

**Affiliations:** 1 Institute for Microtechnology, TU Braunschweig, Alte Salzdahlumer Str 203, 38124 Braunschweig, Germany; E-Mails: a.phataralaoha@tu-bs.de; s.buettgenbach@tu-bs.de; 2 Institute for Process Measurement and Sensor Technology, TU Ilmenau, Gustav-Kirchhoff-Str. 1D-98693 Ilmenau, Germany; E-Mails: norbert.hofmann@tu-ilmenau.de; gerd.jaeger@tu-ilmenau.de

**Keywords:** Force sensors, diffused piezoresistors, swastika membrane, 3D measurements, NPMM

## Abstract

In this contribution, we report on different miniaturized bulk micro machined three-axes piezoresistive force sensors for nanopositioning and nanomeasuring machine (NPMM). Various boss membrane structures, such as one boss full/cross, five boss full/cross and swastika membranes, were used as a basic structure for the force sensors. All designs have 16 p-type diffused piezoresistors on the surface of the membrane. Sensitivities in *x, y* and *z* directions are measured. Simulated and measured stiffness ratio in horizontal to vertical direction is measured for each design. Effect of the length of the stylus on H:V stiffness ratio is studied. Minimum and maximum deflection and resonance frequency are measured for all designs. The sensors were placed in a nanopositioning and nanomeasuring machine and one point measurements were performed for all the designs. Lastly the application of the sensor is shown, where dimension of a cube is measured using the sensor.

## 1. Introduction

Silicon tactile sensors have been developed for metrology, robotics and biomedical applications. For example, three-axes force sensors can be used to characterize geometry and mechanical properties of micro structures [1]. Robot fingers integrated with force sensors can perform complex grasping tasks [2]. A surgical cutting tool has been connected to a three-axes force sensor for accurate force sensing in fetal surgery [3].

There are several approaches to the design and fabrication of force sensors. Two and three dimensional cantilever and capacitive [4] force sensors have been studied in detail in the past. Cantilevers can measure only in 2 D, whereas capacitive force sensors are very complex due to the compulsory electronic circuits for capacitance detection. There are other optical- and laser-based measurements on multi-sensor systems which provide highly accurate measurement on visible surfaces, but it is difficult to use these systems for 3D measurements. This is an important reason why many groups are working on developing a tactile sensor using a four arm structure [5-8] for contact probing.

As far as the state of the art of 3D silicon-based force sensors is concerned, they have been developed mainly using the piezoresistive and capacitive sensing principles. Chu *et al.* [9] reported on a 3D tactile sensor based on the differential capacitive principle, where the measuring range and the sensitivity could be adjusted by changing the membrane's thickness. Fabrication is quite complicated due to combination of elastomer, silicon, glass and polymer, which is simplified in this work by only using silicon. There is offset in output signal due to anodic bonding used in the fabrication, also the cross talk cannot be neglected, because four electrodes are used.

Recently, a 3D force sensor has been fabricated using a titanium foil, where a stylus is attached to the centre of the symmetrical four-arm titanium foil structure [5]. The drawback is that the strain gauges are individually glued onto the titanium foil which leads to variations in the position of the strain gauges on the foil. Strain gauge position variations lead to sensitivity variation from sensor to senor. Here, the strain gauges are diffused in the membrane thus positioning uncertainty is almost negligible.

In this work, we have simulated, fabricated and characterized miniaturized three-axes piezoresistive force sensors with 16 p-type piezoresistors on the boss membrane structure, which are connected in a Wheatstone bridge form. The sensitivity of the sensors can be enhanced by optimally designing the membrane structure. We have fabricated and characterized five different membrane designs.

Here, various results for e.g. sensitivity in *x, y* and *z* direction, maximum and minimum deflections and resonance frequency of each designs are measured and summarized. Simulations were performed by varying the length of the stylus to study its impact on H:V stiffness ratio and deflection in the membrane. The sensors were integrated in nanopositioning and nanomeasuring machine (NPMM) for measurements and characterization of the sensors. An application of the 3D force sensor is presented, where it is mounted in a NPMM for dimensional measurement of a cube.

## 2. Working principle of the 3D force sensor

**Figure 1.**
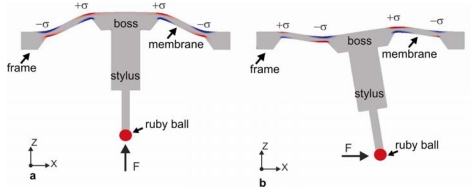
Schematic of deformed shape of the membrane for applied force a) *F_Z_*, b) *F_X_* or *F_Y_*.

The working principle of the three-axes force sensor can be explained as follows. The boss membrane consists of three parts, namely a boss, a membrane and a frame. A stylus is glued at the centre of the boss, which is 5 mm long and has a ruby ball which is 300 μm in diameter attached at the end. Depending upon the direction of the applied force, the membrane has two kinds of important deformation mode. Schematic drawings of deformed shapes of the membrane structure are used to describe the working principle of the force sensor (Figure 1).

When a vertical force (*z* direction) is applied on the stylus, the membrane deflects vertically up or down (Figure 1a). When a lateral force is applied on the stylus (*x* or *y* direction), the membrane is being twisted around the *x* or *y* axes according to the applied force. Piezoresistors in the form of Wheatstone bridges are placed on the membrane at the locations where maximum strain is generated. Due to the strain in the membrane the resistivity of the p-diffused piezoresistor changes. The mechanical signal can be converted into electrical signals by using the imbalance of excited Wheatstone bridge circuits.

## 3. Membrane designs for the force sensors

**Figure 2.**
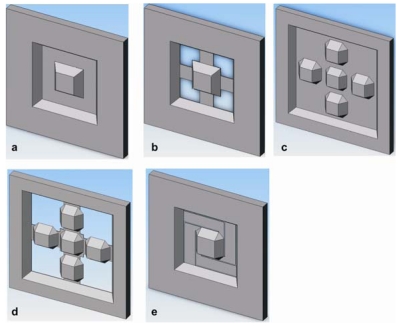
Different membrane designs a) full, b) cross, c) five boss full, d) five boss cross and e) swastika membrane.

Properties of the sensor such as sensitivity, stiffness and deflection can be adjusted by varying the membrane design. For e.g. a cross membrane gives higher sensitivity compared to full membrane, because the stiff edges are through etched, leading to higher deflection. Different applications require different stiffness i.e. –for dynamic measurements the membranes should have high stiffness, whereas for static it should have less stiffness. For simplicity of electronic hardware it is better to have same stiffness in the *x, y* and *z* directions. Depending upon the application one can choose a membrane design. In this work, five membrane designs are fabricated, simulated and characterized (Figure 2). Figures 2a and b shows the conventional one boss full and cross membrane. To get comparable stiffness in the *x, y* and *z* directions many groups are concentrating on modifying the boss membrane design by using eight beams [10], twin membranes [11] and five boss cross membranes (CM) fabricated using dry etching [12].

For comparison with other designs, we fabricated five boss full and cross membrane design using wet etching (figure 2c and d). The five boss membrane are intended for dynamic measurements, which requires high stiffness. Lastly, a long L-shaped membrane, which we call ‘swastika’, is fabricated to get high deflections. Dimensions of all the five designs are summarized in the Table 1. For all the five designs the total chip size remains constant at 6.5 × 6.5 mm². The frame size for five boss designs are smaller compared to other designs to adjust the additional bosses. Along with other dimensions, resistor dimensions are also summarized in Table 1, which are placed at maximum strain locations on each membrane i.e. near the edge of the boss or/and the frame. For swastika membrane the piezoresistors are at the edge of the frame on all four L-shaped membrane.

## 4. Fabrication and measurement set-up

The boss membranes were fabricated using 〈100〉 n-type wafers with a thickness of 360 ± 25 μm and with a background resistivity of 3-6 Ωcm. The strain gauges are aligned with the crystal directions 〈110〉 and 〈110̄〉 of (100) silicon, where coefficient of p-piezoresistivity reaches its maximum value.

The n-Si was oxidized and structured using photolithography to provide opening for piezoresistors i.e. for p-diffusion. p-diffusion was then performed by spinning Borofilm 100^®^ on the substrate and heating it at 900 °C (sheet resistivity = 55 Ω/square, concentration = 1.8 × 10^19^ cm^-3^, depth = 0.6 μm) followed by second lithography step for p^+^-diffusion to get ohmic contacts. The sheet resistance of the p^+^‐doped lines is around 4 Ω/square. Aluminium was then sputtered and structured for contacts, which was tempered at 400 °C in the wet atmosphere. Silicon nitride was deposited on the backside using PECVD and structured to etch the membrane using KOH. The membrane was etched until a thickness of approximately 25 μm was achieved. For fabricating a cross membrane the last step is dry etching, which was performed using inductive plasma.

**Figure 3.**
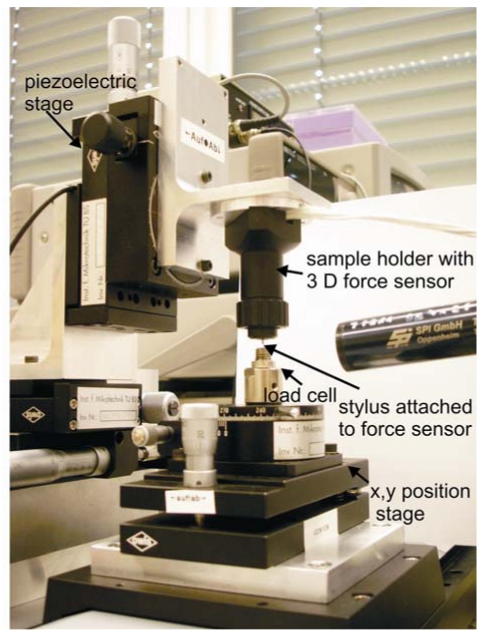
Set-up used for the measurements.

A photograph of the measurement set-up is shown in Figure 3. The set-up consists of a *x, y* position stage, load cell, sample holder and a piezoelectric stage. The position stage is used to fine position the load cell. The sample holder is used to place the samples, which can be tilted in vertical and horizontal direction, due to which force can be applied on the stylus in *x, y* and *z* directions. The sample holder is designed and manufactured in IMT, Braunschweig. A precision piezoelectric stage (from OWIS) is used for deflecting the stylus. The set-up allows a maximum deflection of 25 μm with a resolution of 50 nm. The sample holder along with sample is mounted on the piezoelectric stage and is moved downward under a computer controlled program. A commercial load cell (from ME-Meβsysteme GmbH) is used to measure the probing force. A stabilized voltage source and amplifier (from Dewetron) is applied to the bridge circuits and change of offset voltage due to applied force is measured and collected by a computer controlled programme for all the four bridges.

## 5. Experimental and simulated results

### 5.1. Stiffness ratio

The silicon tactile microprobe consists of a boss-membrane structure with a membrane thickness of approximately 25 μm fabricated by anisotropic wet chemical etching process. As tactile element, a 5 mm long stylus with a probing ball at its end is fixed in the middle of boss (see Figure 1). Force can be applied vertically (Figure 1a) or laterally (Figure 1b) on the tactile element, which then leads to membrane deformation.

**Figure 4.**
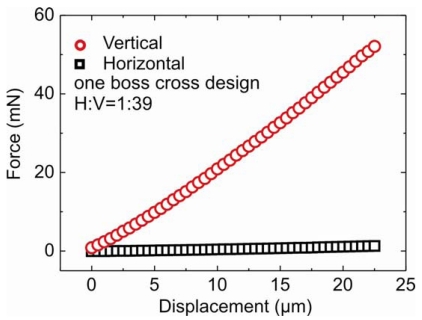
Force vs displacement plot for one boss cross membrane.

Horizontal to vertical (H:V) stiffness ratio of all the five designs were simulated using COSMOS software which is used for structural research and analysis. To avoid the hardware complexity the H:V ratio should be ideally equal to one. Measured plot of force and displacement is shown in figure 4 for one boss cross design. A H:V stiffness ratio of 1:39 is obtained, whereas the simulation gives a ratio of 1:40. Similar plots were obtained for other designs. The simulations were performed at minimum and maximum actual thickness membrane and error bars are calculated. The membrane thickness for all the designs are tabulated in Table 1. Practically, stiffness of several sensors were measured. The average results along with error bars are presented in Table 2 along with the simulated results. The simulated and measured results are comparable to each other. For five boss cross designs fabricated using dry etching H:V ratio of 1:4 is obtained (see Ruther *et.al* [13]). For the same design fabricated using wet-etching we obtain H:V ratio of 1:2.28 ± 0.74. Differences are visible in one and five boss full membrane. The differences could be due to the uncertainty in positioning the stylus at the centre of the boss during measurements.

Compared to work done by other group, the four arm titanium foil sensor gives H:V ratio of 1:15 [5], which is lower then one boss full and cross membrane.

To understand the effect of the length of the stylus on H:V stiffness ratio and on the deflection of the membrane, simulations were performed on a one boss full membrane with a stylus of different length. The membrane thickness was kept constant at 25 μm. It was observed that the H:V stiffness ratio decreases to 1:20.8 from 1:47 when the stylus is 3 mm long instead of 5mm. On the other hand, the ratio increases to 1:77 at 7 mm length. The length of the stylus plays a crucial role in controlling the H:V stiffness ratio, hence should be chosen carefully depending on the application. It was also observed that the horizontal stiffness increases with the decrease in the stylus length, leading to lower deflection. Vertical stiffness is not dependent on the length of the stylus, which is expected behaviour.

### 5.2. Sensitivity

The sensitivity of the sensor is dependent on the membrane structure. The sensitivity can be measured in *x, y* and *z* directions. To measure the sensitivity of the sensor a constant input voltage of 1 V is applied across the Wheatstone bridge. Ideally all the diffused piezoresistors of the Wheatstone bridge are equal, and hence the offset voltage (referred to as output signal) should be zero. However, there are always some non-ideal circumstances that cause a small non-zero output signal, which had to be set to zero before starting the measurements.

**Figure 5.**
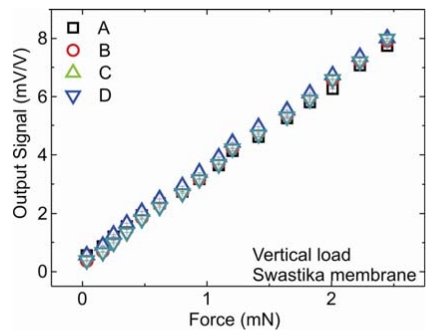
Output voltage vs force plot for swastika membrane.

The sensitivity in the *z* direction can be calculated using equation (1):

(1) $$S_{Z}=A+B+C+D$$

where *S_z_* is the transformed signal in *z* direction and *A-D* are the output signals of the four Wheatstone bridges. Figure 5 shows the plot for output voltage vs force for swastika membrane under vertical force. Output signal increases with the increase in the force. Plots for all the force channels are linear. The vertical sensitivity of swastika membrane calculated using equation one is 12.28 ± 0.09 mV/V/mN.

The sensitivity in *x* or *y* direction can be calculated using the equations below:

(2) $$S_{X}=A-C$$

(3) $$S_{Y}=B-D$$

where *S_X_* and *S_Y_* are the transformed signal in the *x* and *y* directions, respectively.

The vertical and horizontal sensitivity for all the five designs are summarized in Table 3. The highest vertical sensitivity is given by swastika membrane and horizontal by one boss cross membrane. Unfortunately we are unable to measure horizontal sensitivity of swastika membrane using our set-up (described above), because the swastika membrane is more sensitive than the load cell.

### 5.3. Minimum-maximum deflection and resonance frequency

A break test was performed on the membranes to estimate maximum horizontal and vertical displacement the membranes can withstand. The results for all the five designs are tabulated in Table 4.

The cross-shaped membrane breaks at higher force compared to full membrane in vertical direction. Cross-shaped membrane can deflect more in vertical direction, due to less stiffness, compared to the full membrane. In lateral direction, both cross-shaped and full membranes break at same force, because the stiffness of both the membranes in this direction is comparable. Five boss full and cross membranes have comparable stiffness in both horizontal and vertical direction, resulting in comparable highest deflection. As expected, the swastika membrane gave highest deflection in both directions, due to its long length. It was observed that the stylus doesn't unglue itself nor does it break, which is to be expected, because the stylus and glued surface is much stiffer than the membranes.

The minimum detectable force or deflection is defined by the noise in the system. The value of noise (3σ) is equal to 11.52 μV. Using the 3σ and the measured sensitivity, the minimum force and displacement can be calculated, which is 9 μN and 1.4 nm, respectively. In comparison to the result of four armed titanium force sensor (160 μN and 840 nm) [5], our sensors have 18 times higher accuracy for force measurement and 600 times for the displacement measurement.

Another important parameter of the sensor is the resonance frequency, which is measured in horizontal and vertical direction. During the scanning measurements the sensor vibrates and therefore the resonance frequency of the sensor should be much higher then this vibration frequency. The resonance frequency for all the five designs are measured and are tabulated in Table 5. Four arm titanium foil force sensor has resonance frequency of 417 Hz [5], which is comparable to one boss cross membrane.

### 5.4. Characterization and Application using NPMM

All sensors were integrated in a nanopositioning and nanomeasuring machine (NPMM) [14]. The NPMM allows measurements in a range of 25 mm x 25 mm x 5 mm with a resolution of 0.1 nm [15]. The sensor is fixed on the metrology-frame on the NPMM, while the specimen is placed on a movement stage.

**Figure 6.**
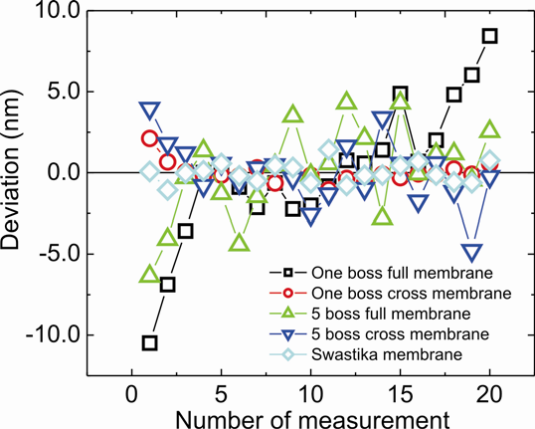
Deviation vs number of measurements for single point measurement for all the membrane design.

Figure 6 shows the result of a single point measurement for all membrane types. It shows the deviation of the position of the measured point (in measurement direction) for 20 measurements. The results were achieved for measurements in the *x-y* plane. The standard deviations of the measurements were calculated to obtain reproducibility. The reproducibility was defined as two times standard deviation. The measured reproducibility, that include not only the one of the force sensor but also the one of the NPMM were in a range from 8.6 nm for the one boss full membrane probe and 1.3 nm for the one boss cross membrane probe and the swastika membrane probe. For the five boss full and cross membrane, it is measured 5.7 nm and 4 nm, respectively.

**Figure 7.**
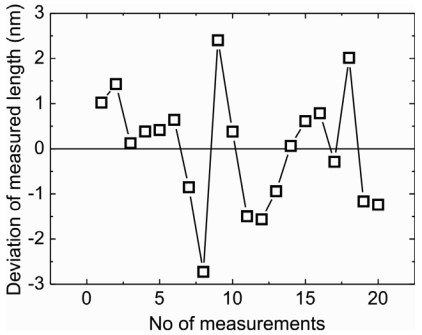
A cube of 10 mm length measured using swastika membrane.

Lastly, an application of the membrane is shown where the sensor is integrated in the NPMM to measure the dimensions of a cube. Deviation of measured length vs no of measurement is shown in Figure 7. The length of the cube was measured to be 10 mm with a very low standard deviation of 1.3 nm.

## 6. Conclusions

3D force sensors were successfully fabricated using silicon bulk micromachining. Various membrane structures were designed to vary the sensor properties such as stiffness, sensitivity, resonance frequency and horizontal to vertical stiffness ratio. Simulated and measured H:V stiffness ratios are comparable. The highest ratio of 0.438 is achieved by a five boss cross membrane. H:V stiffness ratio can be reduced by reducing the length of the stylus. The highest vertical sensitivity of 12.28 ± 0.09 mV/V/mN is achieved using a swastika membrane and highest horizontal sensitivity using a one boss cross design (11.29 ± 0.29 mV/V/mN). Highest vertical and horizontal deflections, which are found to be 140 ± 5 μm and 640 ± 10 μm, respectively, are given by the swastika membrane. The minimum force and displacement that can be detected is 9 μN and 1.4 nm. The highest resonance frequency of 1830 Hz is given by the one boss full membrane and lowest of 214 Hz by the swastika membrane. For single point measurement best reproducibity is achieved to be 1.3 nm for the one boss cross and swastika designs. A cube is successfully measured using the swastika membrane with a very low standard deviation of 1.3 nm.

## Figures and Tables

**Table 1.** Summary of dimensions of five different membrane designs.

**Dimension**	**Full**	**Cross**	**5 boss full**	**5 boss cross**	**Swastika**
Membrane					
l (μm)	700	700	1550	1550	1600
w (μm)	3000	700	4500	1350	600
t (μm)	18 ± 3	25 ± 1	28 ± 2	25 ± 1	25 ± 2
Boss					
l (μm)	1600	1600	1300	1300	1600
w (μm)	1600	1600	1300	1300	1600
Frame					
l (μm)	1750	1750	1050	1050	1750
w (μm)	6500	6500	6500	6500	6500
Resistor					
l (μm)	440	440	440	440	140
w (μm)	20	20	20	20	20

* l is length, w is width and t is the thickness.

**Table 2.** Simulated and measured horizontal to vertical stiffness ratio.

	**Full**	**Cross**	**5 boss full**	**5 boss cross**	**Swastika**
Simulated (membrane thickness= See Table 1)	1:40 ± 2	1:41 ± 1	1:10 ± 1	1:6 ± 0.25	1:26 ± 1
Measured (membrane thickness= See Table 1)	1:25 ± 1	1:36 ± 2	1:19	1:2.28 ± 0.74	-

**Table 3.** Summary of sensitivity in horizontal and vertical direction for all five designs.

**Sensitivity (mV/V/mN)**	**Full**	**Cross**	**5 boss full**	**5 boss cross**	**Swastika**
Vertical	0.70 ± 0.017	3.01 ± 0.036	1.79 ± 0.02	3 ± 0.01	12.28 ± 0.09
Horizontal	2.57 ± 0.04	11.29 ± 0.29	0.1± 0.02	0.26 ± 0.014	-

**Table 4.** Summary of maximum displacement for all membrane designs.

**Maximum displacement (μm)**	**Full**	**Cross**	**5 boss full**	**5 boss cross**	**Swastika**
Vertical	27 ± 3	37 ± 3	90 ± 4	100 ± 2	140 ± 5
Horizontal	150 ± 10	150 ± 10	50 ± 5	60 ± 2	640 ± 10

**Table 5.** Resonance frequency for all the designs.

**Resonance frequency (Hz)**	**Full**	**Cross**	**5 boss full**	**5 boss cross**	**Swastika**
Horizontal direction	1,830	460	-	1,400	214
Vertical direction	4,040	900	-	2,014	327
